# Important Role of a Putative Lytic Transglycosylase Cj0843c in β-Lactam Resistance in *Campylobacter jejuni*

**DOI:** 10.3389/fmicb.2015.01292

**Published:** 2015-11-17

**Authors:** Ximin Zeng, Barbara Gillespie, Jun Lin

**Affiliations:** Department of Animal Science, The University of Tennessee, KnoxvilleTN, USA

**Keywords:** beta-lactamase, peptidoglycan, random transposon mutagenesis, lytic transglycosylase

## Abstract

Beta-lactam antibiotics are an important class of antibiotics for treating bacterial infections. Despite prevalent β-lactam resistance in *Campylobacter jejuni*, the leading bacterial cause of human diarrhea in developed countries, molecular mechanism of β-lactam resistance in *C. jejuni* is still largely unknown. In this study, *C. jejuni* 81–176 was used for random transposon mutagenesis. Screening of a 2,800-mutant library identified 22 mutants with increased susceptibility to ampicillin. Of these mutants, two mutants contains mutations in *Cj0843c* (a putative lytic transglycosylase gene) and in its upstream gene *Cj0844c*, respectively. Complementation experiment demonstrated that the Cj0843 contributes to β-lactam resistance. The *Cj0843c* insertional mutation was subsequently introduced to diverse *C. jejuni* clinical strains for MIC test, showing that Cj0843c contributes to both intrinsic and acquired β-lactam resistance of *C. jejuni*. Consistent with this finding, inactivation of Cj0843c also dramatically reduced β-lactamase activity. Genomic examination and PCR analysis showed *Cj0843c* is widely distributed in *C. jejuni*. High purity recombinant Cj0843c was produced for generation of specific antiserum. The Cj0843 was localized in the periplasm, as demonstrated by immunoblotting using specific antibodies. Turbidimetric assay further demonstrated the capability of the purified Cj0843c to hydrolyze cell walls. Inactivation of Cj0843c also significantly reduced *C. jejuni* colonization in the intestine. Together, this study identifies a mechanism of β-lactam resistance in *C. jejuni* and provides insights into the role of cell wall metabolism in regulating β-lactamase activity.

## Introduction

Beta-lactam antibiotics, which inhibit biosynthesis of bacterial cell wall, are the most commercially available antibiotics in the market. For instance, in 2009, beta-lactam antibiotics account for more than half of the total antibiotic sales globally (Hamad, 2010). However, the efficacy of the β-lactam based therapy is severely threatened by a variety of β-lactam resistance mechanisms in bacteria, such as production of beta-lactamases (Korfmann and Wiedemann, 1988; Iaconis and Sanders, 1990; Jacoby, 2009), expression of the penicillin-binding proteins (PBPs) with reduced affinity for β-lactam antibiotics (Fuda et al., 2004), and extrusion of β-lactam using multi-drug eﬄux pumps (Nikaido, 1998; Vila and Martinez, 2008). Production of β-lactamase has been a major and threatening β-lactam resistance mechanism. Recently, increasing evidence demonstrate a direct link between cell wall metabolism and regulation of β-lactamase activity in Gram-negative bacteria, which provides insights into the development of innovative strategies to counteract β-lactam resistance in pathogenic bacteria (Zeng and Lin, 2013).

*Campylobacter jejuni* is the leading bacterial cause of human gastroenteritis in the developed countries (Humphrey et al., 2007). Clinical symptoms include fever and watery diarrhea in humans. In rare case, campylobacteriosis can cause severe sequelae such as Guillain–Barré syndrome, an acute flaccid paralysis that may lead to respiratory muscle compromise and death (Nachamkin et al., 1998; Hughes and Cornblath, 2005; Nyati and Nyati, 2013). The annual incidence of campylobacteriosis in the United States is approximately 1% (Poly and Guerry, 2008), resulting in 1.7 billion dollars cost in medical practice and productivity (Hoffmann et al., 2012). In parallel to its increased prevalence, *Campylobacter* is increasingly resistant to macrolides and fluoroquinolones, the two major clinical antibiotics (Luo et al., 2005; Caldwell et al., 2008; Koningstein et al., 2011). Recently, it has been proposed that an oral β-lactam, such as co-amoxiclav, may provide an alternative therapy for *Campylobacter* infection (Elviss et al., 2009; Griggs et al., 2009). However, β-lactam resistant *C. jejuni* strains have also been observed in epidemiological studies (Lachance et al., 1991; Griggs et al., 2009). Thus, understanding the mechanisms of β-lactam resistance in *C. jejuni* would help us develop effective therapeutic strategy that will allow the use of existing β-lactams against resistant *C. jejuni*. In *Campylobacter*, two beta-lactam resistance mechanisms have been characterized, which include the presence of CmeABC multi-drug eﬄux pump (Lin et al., 2002) and the production β-lactamase OXA-61 (Alfredson and Korolik, 2005; Griggs et al., 2009). Despite these progresses, the findings from recent studies strongly suggest the existence of novel β-lactamases and/or regulatory system in *C. jejuni* for β-lactam resistance (Griggs et al., 2009; Zeng et al., 2014).

In this study, using random mutagenesis, we identified a putative lytic transglycosylase (LT) Cj0843c that is required for intrinsic and acquired β-lactam resistance in *C. jejuni*. This finding was further validated by a series of molecular, biochemical and genomic examination. Consistent with this finding, inactivation of Cj0843c, which is highly conserved in *C. jejuni*, dramatically reduced β-lactamase activity. The role of Cj0843c in peptidoglycan metabolism was validated by a standard LT assay using purified Cj0843c. This study provides compelling evidence that Cj0843c, a putative LT, plays a critical role in regulating β-lactamase-mediated β-lactam resistance in *C. jejuni*.

## Materials and Methods

### Bacterial Strains, Plasmids, and Culture Conditions

The major bacterial strains and plasmids used in this study are listed in **Table 1**. The *C. jejuni* strains were grown routinely in Müller-Hinton (MH) broth or agar at 42°C in tri-gas incubator (85% N_2_, 10% CO_2_, 5% O_2_). *Escherichia coli* strains were grown routinely in Luria-Bertani (LB) broth with shaking (250 rpm) or on agar at 37°C overnight. When needed, culture media were supplemented with ampicillin (100 μg/ml), kanamycin (50 μg/ml), and chloramphenicol (Cm; 20 μg/ml).

**Table 1:** Key bacterial plasmids and strains used in this study.

Plasmids or strains	Description	Source or reference
**Plasmids**		
pRY111	*Escherichia coli–Campylobacter* shuttle vector, kanamycin resistant	Yao et al., 1993
pCj843c	pRY111 derivative containing *Cj0843c, Cj0844c*, and *Cj0845c*	This study
pCj844c	pRY111 derivative containing *Cj0843c* and *Cj0844c*	This study
pET28b-Cj0843c	Expression vector for recombinant Cj0843c, Kan^r^	Novagen
**Strains**		
*Campylobacter jejuni*		
JL241	NCTC 11168, human isolate	Parkhill et al., 2000
JL890	JL241 derivative with transposon inserted into *Cj0843c*, Kan^r^	This study
JL990	JL890 derivative, complemented with pCj843c	This study
JL28	81–176, human isolate, for random transposon mutagenesis	Black et al., 1988
JL30	81–176 (JL28) derivative with transposon inserted into *cmeB* gene, Kan^r^	Lin et al., 2002
JL916	JL28 derivative with EZ::TN <KAN-2> transposon inserted into *Cj0843c*, Kan^r^	This study
JL926	JL916 derivative, complemented with pCj843c	This study
JL962	81–176 derivative, ampicillin resistant. The β-lactamase gene *bla*_OXA-61_ was introduced into chromosome via natural transformation using genomic DNA from ampicillin resistant *C. jejuni* 21190 (Zeng et al., 2014)	This study
JL963	JL962 derivative. The *Cj0843c::kan* mutation was introduced into chromosome via natural transformation using genomic DNA from JL916, Kan^r^	This study
JL995	81–176 (JL28) derivative, Kan^r^. The *pgp1::kan* mutation was introduced into JL28 chromosome via natural transformation using genomic DNA of the *pgp1* mutant (Frirdich et al., 2012)	This study
JL996	JL962 derivative. The *pgp1::kan* mutation was introduced into JL962 chromosome via natural transformation using genomic DNA of the *pgp1* mutant (Frirdich et al., 2012)	This study
*E. coli*		
DH5α	F- Φ80*lacZ*ΔM15 Δ(*lacZYA-argF*)U169 *recA1 endA1 hsdR17* (rk-, mk+) *phoA* supE44 thi-1 *gyrA96 relA1* λ-	Invitrogen
JL985	pET28b-Cj0843c in BL21(DE3)	This study


### Random Transposon Mutagenesis

We have successfully used *C. jejuni* 81–176 as a host strain for *in vivo* random transposon mutagenesis using EZ-Tn5 < KAN-2 > Tnp Transposome system (Epicentre; Lin et al., 2009; Hoang et al., 2011, 2012; Zeng et al., 2013b). In this study, the wild-type *C. jejuni* 81–176 (ampicillin Minimum inhibitory concentration (MIC) = 1 μg/ml) was subjected to *in vivo* random transposon mutagenesis; the procedure and screening strategy are detailed in previous publications (Lin et al., 2009; Hoang et al., 2011, 2012; Zeng et al., 2013b). The mutants with growth defects in MH broth containing kanamycin (50 μg/ml) and ampicillin (0.25 μg/mL) were identified. To determine transposon insertion site, genomic DNA extracted from the selected mutant was subjected to direct sequencing as described previously (Lin et al., 2009; Hoang et al., 2011; Zeng et al., 2013b).

### Antimicrobial Susceptibility Test

The susceptibilities of *C. jejuni* strains to different antimicrobials (Fisher Bioreagents) were determined by a standard microtiter broth dilution method with an inoculum of 10^6^ CFU/ml as described previously (Lin et al., 2002; Zeng et al., 2014). MIC for specific antimicrobial was defined by the lowest concentration of the antimicrobial showing complete inhibition of bacterial growth after 2 days of incubation at 42°C under microaerophilic condition.

### Beta-lactamase Activity Assay

To measure β-lactamase activity in *C. jejuni* strains, standard assay was performed on cell lysates of different strains by following previous procedure (Griggs et al., 2009; Zeng et al., 2014) with minor modifications. Briefly, late-log phase bacterial cells were harvested from MH plates, washed with PBS, and adjusted to an optical density at 600 nm of approximately 1.2 in PBS. Bacterial suspensions were sonicated and centrifuged at 13,000 rpm for 5 min. The supernatant was subjected to protein concentration measurement using BCA Protein Assay kit (Pierce). One hundred microliter of supernatant containing 40 μg of protein was mixed with 0.8 ml of ice-chilled reaction buffer (0.1 M phosphate; 1 mM EDTA, pH 7.0). Then 100 μl of nitrocefin (500 μg/ml) was added to the above reaction mix followed by incubation at 37°C for 30 min. The activity was determined by spectrometric at 486 nm. The molar extinction coefficient of hydrolyzed nitrocefin at 486 nm is 20,500 M^-1^ cm^-1^. The lysate from *C. jejuni* 81–176 was used as negative control. The β-lactamase activity was expressed as hydrolyzed nitrocefin (μM) per hr per mg of protein.

### Complementation of *C. jejuni* 81–176 Transposon Mutants

The long fragment (containing the genes *Cj0843c*, *Cj0844c*, and *Cj0845c*) and the short fragment (containing the genes *Cj0844c* and *Cj0845c*) were PCR amplified from *C. jejuni* 81–176 using primer pairs of Cj0843c_F/Cj0843c_R and Cj0843c_F/Cj0844c_R (**Table 2**), respectively, with *Pfu*Ultra fusion II DNA polymerase (Stratagene). The resulting fragments were ligated into *Sma*I-digested shuttle vector pRY111 (Yao et al., 1993) and ligation mix was introduced into *E. coli* DH5α by heat shock transformation. The *E. coli* transformants containing the plasmid that bears the three-gene (named pCj843c) or the two-gene fragment (named pCj0844c) were identified and confirmed by sequencing. Both constructs were mobilized into *C. jejuni* 81–176 transposon mutants by triparental conjugation as described previously (Zeng et al., 2013a, 2014).

**Table 2:** Major primers used in this study.

Primer	DNA Sequence (5′–3′)^a^	Product size (bp)	Target gene and function
Cj0843c_F	AACAAAATCCCAAGCTAAAGTCA	3,767	*Cj0843c, Cj0844c* and *Cj0845c* operon
Cj0843c _R	TTCTAGACAAGACAAGTAAAGATGATG		
Cj0844c_R	CAAGTGAATTTTCTTCCTTTTTGAG	2,028	With Cj0843c_F, to amplify *Cj0844c* and *Cj0845c*
Cj0843c_F1	GCTCAAACCTTTAAAAATCAAAGA	586	*Cj0843c* prevalence and validation of *Cj0843c* mutation
Cj0843c_R1	TTTGCCAAGCAAAAGGATCT		
rCj0843c_F1(NdeI)	GGAATTCCATATGCAATATAGTATAGAAAAACTCAAAAAGGAA	1,568	Recombinant Cj0843c
rCj0843c-pET28b_R(SalI)	GCGCATGTCGACCTAAGATTTGTTTAGATCATTTGCC		


*^a^Restriction sites are underlined in the primer sequence and the names are identified in parentheses.*

### Natural Transformation

Natural transformation (biphasic method) was performed following standard procedure as described by Wang and Taylor (1990). Approximately 4 μg of *C. jejuni* genomic DNA was used for natural transformation. The insertional *Cj0843c* mutations in different strains were confirmed by PCR using primer pair Cj0843c_F1 and Cj0843c_R1 (**Table 2**).

### Production of Recombinant Cj0843c and Generation of Polyclonal Antiserum

N-terminal histidine-tagged recombinant Cj0843c was produced in *E. coli* by using pET-28b(+) vector. Briefly, approximately 1568-bp fragment of *Cj0843*c was PCR amplified from *C. jejuni* NCTC 81–176 using primer pairs rCj0843c_F1(*Nde*I) and rCj0843c-pET28b_R(*Sal*I; **Table 2**). The amplified product and the vector pET-28b(+) were both digested with *Nde*I and *Sal*I and ligated with each other. Cloning, expression, and purification of rCj0843c were performed using the procedures described in our previous publication (Lin et al., 2005; Zeng et al., 2013a). The plasmid pET28b-Cj0843c in the *E. coli* BL21(DE3) clone (JL985) producing rCj0843c was sequenced to confirm the correct insertion. The fractions containing pure rCj0843c were pooled and dialyzed against 1 x PBS buffer (Fisher Bioreagents). The concentration of the purified rCj0843c was determined using BCA Protein Assay Kit (Pierce^TM^). Approximately 4 mg of highly purified rCj0843c was used for the production of rabbit polyclonal antisera by Pacific Immunology Corp (Ramona, CA). Pre- and post-immune serum samples were analyzed by immunoblotting against pure rCj0843c to confirm the generation of specific Cj0843c antibody.

### Enzymatic Activity of rCj0843c

The LT activity of rCj0843c was evaluated using turbidimetric assay as reported before with minor modification (Caldentey and Bamford, 1992). In brief, the isogenic *Cj0843c* mutant of *C. jejuni* 81–176 was harvested from three overnight-cultured MH agar plates and resuspended in 50 ml Tris-HCl buffer (50 mM, pH 7.5). Then 3 ml of chloroform was added in the suspension, which was kept at room temperature for 30 min with gently shaking. Subsequently, the chloroform-extracted cells (the crude cell walls) were pelleted by centrifugation at 13,000 rpm for 5 min, washed once with 50 mM Tris-HCl buffer (pH7.5), and resuspended in 50 ml of 50 mM Tris-HCl buffer (pH 7.5).

To perform turbidimetric assay, the crude cell walls were adjusted to final OD_450__nm_ of approximately 0.45 using 50 mM Tris-HCl buffer. Then the purified rCj0843c was added to 1 ml of such cell wall suspension with final concentration of 24 μg/ml; the cell wall suspension containing PBS instead of rCj0843c served as control. The decrease in turbidity at 450 nm was determined after 2.5 h of incubation at 37°C. The turbidimetric assay was performed with triplicate and the statistical analysis of a Student’s t test was carried out using SAS (v9.4). A *P*-value less than 0.05 was considered significant.

### Localization of the Cj0843c

To determine cellular localization of Cj0843c, the periplasmic and spheroplastic fractions were prepared using the PeriPreps^TM^ Periplasting Kit (Epicentre) as detailed in our recent publication (Zeng et al., 2013a). Different cellular fractions were subsequently subjected to SDS-PAGE and immunoblotting analysis as described in our previous publications (Lin et al., 2002; Zeng et al., 2009, 2013a). Briefly, the protein samples were separated by SDS-PAGE with a 12% (wt/vol) polyacrylamide separating gel. After SDS-PAGE, proteins in the gels were then electrophoretically transferred to nitrocellulose membranes (Bio-Rad) at 60 V for 1 h at 4°C. The membranes were incubated with blocking buffer (5% Nestle skim milk powder in PBS) for 16 h at 4°C prior to incubation with primary antibodies (Rabbit anti-Cj0843c at 1:5000 dilution in the blocking buffer). To ensure the quality of periplasmic and spheroplastic fractions, antibodies directed against CmeR (cytoplasmic control, 1:2000 dilution) were also used as primary antibodies. After incubation at 25°C for 1 h, the blots were washed three times with PBS containing 0.05% Tween 20 and subsequently incubated with secondary antibodies (1:50,000 dilution of goat anti-rabbit IgG-HRP; Kirkegaard and Perry) at 25°C for 1 h. After washing, the blots were developed with the SuperSignal West Dura Chemiluminescent Substrate (Thermo Scientific). Prestained molecular weight markers (Bio-Rad) were coelectrophoresed and blotted to allow estimation of the sizes of the proteins.

### Prevalence of Cj0843c in *C. jejuni*

Total 30 *C. jejuni* strains from different hosts and geographically diverse areas were grown microaerophilically to late exponential phase and harvested by centrifugation. The cell pellets were suspended in 100 μL of water and lysed by boiling for 5 min. After centrifugation, the supernatants were used as template in PCR assay with highly *Cj0843c*-specific primers Cj0843c_F1 and Cj0843c_R1 (**Table 2**). For bioinformatic analysis of *Cj0843c* prevalence among available genome sequences, total 103 *C. jejuni* genomes were extracted from Integrated Microbial Genomes^1^ and the multi-sequence alignment was analyzed using Clustal Omega^2^. In addition, presence of *Cj0843c* homolog was also examined in the genomes of 49 *Campylobacter coli* and 298 *Helicobacter pylori* strains.

### Chicken Colonization Experiment

*Campylobacter jejuni* 81–176 wild-type strain together with its isogenic *Cj0843c* mutant (JL916) and the complementation strain (JL926; **Table 2**) were used for colonization assay in a chicken model system as described in previous studies (Lin et al., 2002; Lin and Martinez, 2006; Hoang et al., 2011; Zeng et al., 2013a). The chicken study was approved by the Institutional Animal Care and Use Committee at The University of Tennessee (Protocol 1387 under oversight of the IACUC Chair Carla Sommardahl). Briefly, 1-day-old broiler chickens were obtained from a commercial hatchery (Hubbard Hatchery, Pikesville, TN, USA). The chickens were negative for *Campylobacter* as determined by culturing cloacal swabs prior to use in this study. Three groups of 4-days-old chickens (10 birds/group) were inoculated with different bacteria via oral gavage using a dose approximately 10^5^ CFU of fresh late log-phase *C. jejuni* per bird. Notably, the motility of parent strain and its *Cj0843c* mutant was confirmed to be at a comparable level prior to challenge. The isogenic *Cj0843c* mutant displayed reduced growth in MH broth when compared to its parent strain. However, the fresh *C. jejuni* cultures with similar growth phase, reflected by the final OD_600__nm_ measurement, were used to challenge chickens; the bacteria in such cultures were assumed to be in the same physiological state. For each group, 5 birds were euthanized and cecal samples were collected at 6 and 10 days post-inoculation. The cecal contents from each bird were weighed and diluted in MH broth. The diluted samples were plated onto MH agar plates with *Campylobacter* specific selective supplements (Oxoid, UK). The number of CFU per gram of cecal contents was calculated for each chicken and was used as an indicator of the colonization level. The detection limit of the plating methods was 10^2^ CFU/g of cecal contents. A bird from which no *Campylobacter* colonies were detected was assigned a conservative value of 99 CFU/g of cecal contents for the purpose of calculating means and for statistical analysis. One-way analysis of variance (ANOVA) followed by a least-significant difference test was used to analyze the significant differences in colonization level (log transformed). A *P*-value less than 0.05 was considered significant.

## Results

### Cj0843c, a Putative Lytic Transglycosylase, is Required for Ampicillin Resistance

The *C. jejuni* 81–176 strain was used as a host strain to generate a library consisting of 2,800 Tn5-insertional mutants. Individual mutant grown in microtiter plates was replicated into screening plates containing MH broth with sublethal concentration of ampicillin (0.25 μg/ml). A total of 22 mutants displayed increased susceptibility to ampicillin. The insertional sites of these mutants were mapped by direct sequencing. It’s not surprising that most of them (20 mutants) contains transposon insertion in *cmeABC* locus which encodes CmeABC multidrug eﬄux pump system which was previously shown to be involved in ampicillin resistance (Lin et al., 2002, 2003). Specifically, transposon insertional mutations were identified in *cmeA* (two different sites with four mutants), *cmeB* (two different sites with four mutants), and *cmeC* (2 different sites with 12 mutants). Interestingly, the other two mutants have transposon insertion identified in two adjacent genes, the CJJ81176_0859 and CJJ81176_0860 (hereinafter, the corresponding universal locus names in *C. jejuni* NCTC 11168, *Cj0843c* and *Cj0844c*, were used). The Cj0843c was annotated as a putative secreted LT. Specifically, Cj0843c contains a highly conserved domain (COG0741, E-value is 3.53e-27) represented by MltE, an endo-specific LT from *E. coli* that catalyzes hydrolysis of glycan chains to generate short chains with 1,6-anhydro-MurNAc ends (Fibriansah et al., 2012). *Cj0844c* was annotated as putative integral membrane protein. Both mutants displayed fourfold MIC reduction for ampicillin when compared to that of wild type 81–176 (**Table 3**).

**Table 3:** Susceptibilities of two representative *C. jejuni* strains and their isogenic mutants to ampicillin.

Parent strain	MIC (μg/mL)					
	
	Wild type	*Cj0843c^-^*	*Cj0844c^-^*	*Cj0843c^-^*/pCj0843c	*Cj0844c^-^*/pCj0843c	*Cj0844c^-^*/pCj0844c	*CmeB^-^*
81–176	1	0.25	0.25	1	1	0.25	0.0625
NCTC 11168	16	2	ND^a^	16	ND	ND	4


*^a^ ND, not determined.*

Genome analysis showed that the start codon of *Cj0843c* overlapped the stop codon of *Cj0844c* by 4 bp nucleotides. In addition, there is only 2 bp nucleotide gap between the start codon of *Cj0844c* and the stop codon of its upstream gene *Cj0845c*. Therefore, the *Cj0845c*, *Cj0844c*, and *Cj0843*c may form an operon. The transposon insertion into *Cj0844c* may cause polar effect, affecting the expression of downstream gene *Cj0843c*. To test this, two constructs with different length (pCj0843c and pCj0844c) was constructed for complementation (**Table 1**). The pCj0843c covers the whole three-gene operon while pCj0844c only contains two upstream genes *Cj0845c* and *Cj0844c*. As shown in **Table 3**, complementation with pCj0843c restored the MIC of both mutants to the level of wild type strain. However, complementation of the *Cj0844c* mutant with pCj0844c failed to restore MIC of ampicillin to wild-type level, indicating that the Cj0843c, not *Cj0844c*, is involved in ampicillin resistance in *C. jejuni* 81–176.

To examine the role of Cj0843c in ampicillin resistance in different strains, the *Cj0843c* transposon mutation was also introduced into *C. jejuni* NCTC 11168 via natural transformation. As shown in **Table 3**, the isogenic *Cj0843c* mutant of NCTC 11168 also displayed increased susceptibility to ampicillin (eightfold reduction) while the MIC of the complementation strain was the same as that of wild type strain.

### Cj0843c is Involved in the Resistance to Various β-lactam Antibiotics in *Campylobacter*

We also examined if inactivation of Cj0843c affected susceptibilities of *C. jejuni* to different β-lactam antibiotics. As shown in **Table 4**, susceptibilities of *Cj0843c* mutant to various β-lactam antibiotics (penicillin G, ticarcillin, carbenicillin, and cloxacillin) significantly increased (fourfold) when compared to wild-type 81–176 strain. Complementation of the isogenic Cj0843c mutant with pCj0843c restored MIC of ampicillin to wild type level (**Table 4**). In terms of other types of antimicrobials, such as bile salts (cholic acid and deoxycholic acid), ciprofloxacin and ethidium bromide (**Table 4**), the susceptibilities of *Cj0843c* mutant did not change when compared to wild-type strain.

**Table 4:** Cj0843 is only involved in *C. jejuni* resistance to various β-lactams.

Chemical	81–176 WT	*cj0843c::Tn5*	*cj0843c::Tn5*/pCj0843c
Penicillin G (μg/ml)	16	4	16
Ticarcillin (μg/ml)	256	64	256
Carbenicillin (μg/ml)	64	16	64
Cloxacillin (μg/ml)	512	128	512
Cholic acid (mg/ml)	12.5	12.5	N/D^a^
Ciprofloxacin (μg/ml)	0.25	0.25	N/D
Deoxycholic acid (mg/ml)	20	20	N/D
Ethidium bromide (μg/ml)	1	1	N/D


*^a^N/D, not determined.*

### Cj0843c also Plays an Important Role in Acquired Ampicillin Resistance

**FIGURE 1:**
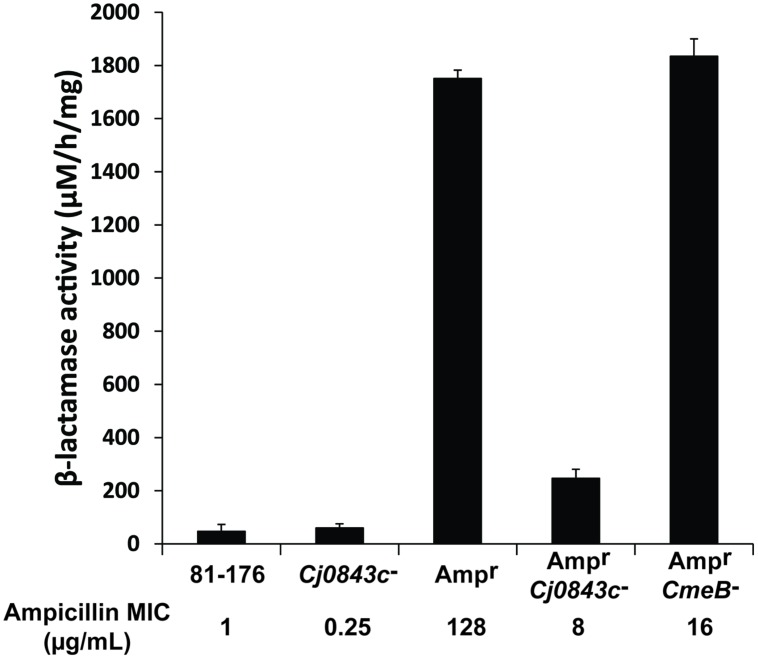
**Effect of *Cj0843c* mutation on intracellular β-lactamase activities and ampicillin resistance in *Campylobacter jejuni*.** The ampicillin MIC values were listed below each corresponding strain. Amp^r^, the 81–176 derivative with β-lactamase gene *bla*_OXA-61_ introduced into chromosome via natural transformation using genomic DNA from ampicillin resistant *C. jejuni* 21190 (Zeng et al., 2014). The procedure for nitrocefin-based β-lactamase activity assay was detailed in Materials and Methods. The activity was determined by spectrometric change at 486 nm. The molar extinction coefficient of hydrolyzed nitrocefin at 486 nm is 20,500 M^-1^ cm^-1^. The β-lactamase activity was expressed as hydrolyzed nitrocefin (μM) per hr per mg of protein.

Although *C. jejuni* 81–176 lacks beta-lactamase gene *bla*_OXA-61_ in the genome (Griggs et al., 2009), it could acquire high-level ampicillin resistance (Amp^r^) due to the acquisition of active beta-lactamase *bla*_OXA-61_ from *C. jejuni* 21190 via natural transformation (Zeng et al., 2014). The MIC of ampicillin for the Amp^r^ derivative of 81–176 was 128 μg/mL, which is 128-fold higher than the MIC of wild-type 81–176 (**Figure 1**), as reflected by the dramatic change of the beta-lactamase activity (**Figure 1**). However, inactivation of *Cj0843c* in the Amp^r^ derivative of 81–176 dramatically reduced MIC (8 μg/mL) as well as the beta-lactamase activity in cell lysates (**Figure 1**). In contrast, although mutation in multidrug eﬄux pump gene *cmeB* (Lin et al., 2002) in the Amp^r^ derivative led to reduced MIC of ampicillin (16 μg/mL), the beta-lactamase activity was not changed (**Figure 1**). This finding clearly indicated that Cj0843c also plays an important role in β-lactamase-mediated ampicillin resistance by modulating the activity of β-lactamase Bla_OXA-61_ in *C. jejuni*.

To determine the role of Pgp1, an identified gene involved cell wall metabolism in *C. jejuni* (Frirdich et al., 2012), in ampicillin resistance, the *pgp1::kan* mutation was introduced into the wild-type 81–176 and its Amp^r^ derivative in this study via natural transformation using genomic DNA of the *pgp1* mutant, creating isogenic *pgp1* mutant JL995 and JL996, respectively (**Table 1**). The MICs for JL995 and JL996 are 1 and 128 μg/mL, respectively, which are the same as those for their corresponding parent strains. Consistent with this finding, the beta-lactamase activity was not changed due to the inactivation of *pgp1*.

### The Prevalent and Highly Conserved Cj0843c is Required for Ampicillin Resistance in Diverse *C. jejuni* Isolates

It’s well known that the genome of *C. jejuni* species displayed considerable plasticity (de Boer et al., 2002; Poly et al., 2008; Duong and Konkel, 2009). In this study, a PCR survey was performed, demonstrating that the *Cj0843c* was present in 30 diverse *C. jejuni* isolates (data not shown). The representative isolates were listed in **Table 5**. Regardless of the level of β-lactamase activity and the level of ampicillin resistance, introduction of *Cj0843c* mutation into these isolates all resulted in the decrease of MIC of ampicillin by more than fourfold (**Table 5**).

**Table 5:** Cj0843c is prevalent and involved in intrinsic and acquired ampicillin resistance in different clinical *C. jejuni* isolates.

Strain	Source/host	*Cj0843c*^a^	β-Lactamase activity^b^	MIC (μg/ml)	
				
				Wild type	*Cj0843c::Tn5*
JL241	Human	+	-	16	4
JL36	Chicken	+	**±**	8	1
JL78	Human	+	-	4	0.25
JL81	Human	+	-	4	0.5
JL85	Human	+	-	2	0.25
JL90	Human	+	-	2	0.5
JL95	Ovine	+	+	16	2
JL100	Human	+	+	16	0.5
JL115	Human	+	++++	256	16
49	Chicken	+	-	4	0.25
Cj41	Chicken	+	**±**	8	1
SO40	Bovine	+	-	16	1
JL98	Human	+	+	8	2
86	Chicken	+	-	16	2


*^a^The prevalence of *Cj0843c* is detected by PCR using primer Cj0843c_F1 and Cj0843c_R1.*

*^b^The β-lactamase activity of each clinical isolate was measured using the cell lysate with the nitrocefin assay (Griggs et al., 2009). A yellow to red color change within 5 min at 37°C indicated a β-lactamase-positive reaction. +, positive β-lactamase activity; -, negative β-lactamase activity; **±**, weak β-lactamase activity; ++++, red color developed immediately upon addition of substrate nitrocefin.*

In addition to this PCR survey, genome analysis also provided compelling evidence showing *Cj0843c* was highly conserved in *C. jejuni* and other closely related species. Specifically, *Cj0843c* was present in all 101 currently available *C. jejuni* genomes deposited in the database. Notably, the three-gene operon containing *Cj0843c* is also highly conserved in the genomes of different 𝜀-proteobacteria, including 101 *C. jejuni*, 49 *C. coli*, and 298 *H. pylori* (**Supplementary Figure S1** and detailed in **Supplementary Table S1**).

The multi-sequence alignment data show the percentage aa identity among *C. jejuni* strains ranges from 93 to 100%. The across-species alignment shows that the aa identity and homology between *C. jejuni* NCTC 11168 and *C. coli* RM2228 are 79 and 89%, respectively, and those between *C. jejuni* NCTC 11168 and *H. pylori* 26695 are 31 and 51%, respectively.

### The Cj0843c is Localized in Periplasm

**FIGURE 2:**
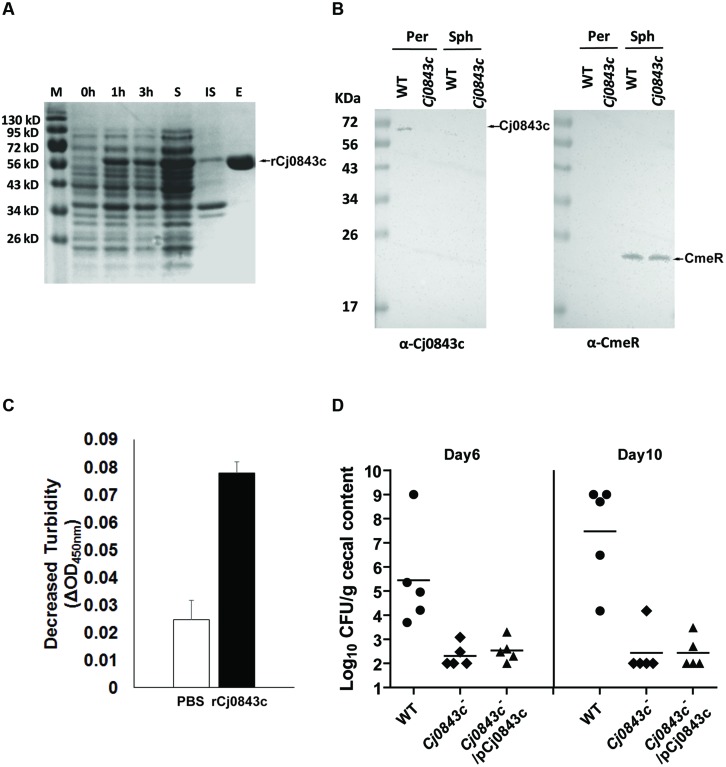
**Characterization of Cj0843c.** **(A)** Expression and purification of rCj0843c. Cj0843 fragment was cloned into pET-28b vector. Expression of rCj0843c was induced in the presence of 0.5 mM IPTG. 0, 1 and 3 h, the cell lysates from pre-induction, 1 and 3 h induced cells; S, soluble fraction; IS, insoluble fraction; E, elution fraction. **(B)** Cj0843c is localized in periplasm. The intracellular regulator CmeR was used as cytoplasm control. Per, periplasm; Sph, spheroplast; WT, wild type. **(C)** Turbidimetric assay of rCj0843c. Chloroform-treated *Cj0843c^-^* mutant cells (crude cell walls) were mixed with rCj0843c or PBS control solution. The decrease of absorbance at 450 nm was measured after 2.5 h incubation. Each bar represents the mean ± SD from three independent measurements. **(D)** Critical role of Cj0843c in colonization of *C. jejuni* in chicken. The chickens in each group (10 birds) were inoculated with *C. jejuni* 81–176 (close circle), its isogenic *Cj0843c* mutant (diamond), and complementation strain (triangle) at dose of 10^5^ CFU/chicken. At days 6 and 10 post-challenge, five chickens from each group were killed and cecal contents were collected for *C. jejuni* numeration. The detection limit is 10^2^ CFU/gram fecal sample. Each horizontal line segment denotes the mean value of log transformed CFU of different strains at the indicated days post-inoculation. The detection limit is 10^2^ CFU/gram fecal sample.

Based on annotation, Cj0843c is predicted to interact with the glycan strand of the periplasmic peptidoglycan. To confirm this, we first cloned *Cj0843c* into expression vector pET-28b and produced N-terminal 6xHis tagged recombinant Cj0843c (rCj0843c). Majority of rCj0843c is in the soluble fraction and was successfully purified by Ni-NTA affinity column (**Figure 2A**).

The specific Cj0843c antiserum was successfully raised and was used for immunoblotting to determine localization of Cj0843c in *C. jejuni*. As shown in **Figure 2B**, Cj0843c is detected in the periplasm fraction but not in the spheroplast fraction of wild-type cells (left panel). The Cj0843c was not detected in the periplasm fraction of the isogenic Cj0843c mutant either (left panel in **Figure 2B**). As a control, CmeR, an intracellular regulator, is only detected in the spheroplast fractions in both wild type and *Cj0843c* mutants (right panel in **Figure 2B**). This immunoblotting analysis provides compelling evidence that that Cj0843c is localized in the periplasm, the place where peptidoglycan resides in Gram-negative bacteria.

### Lytic Transglycosylase Activity of rCj0843c

The chloroform-treated *Cj0843c* mutant cells was used as cell wall substrates because the rCj0843c action sites on cell walls from this mutant are possibly intact. As shown in **Figure 2C**, the decrease in OD_450nm_ (ΔOD_450nm_) in the reaction solution containing the rCj0843c is significantly larger than PBS control (*P* < 0.05), indicating the capability of rCj0843c as LT to hydrolyze cell wall.

### Cj0843c is Required for *C. jejuni* Colonization

The role of Cj0843c in *in vivo* survival of *C. jejuni* was evaluated using chicken model system. As shown in **Figure 2D**, wild-type *C. jejuni* 81–176 could colonize chicken efficiently on days 6 and 10 post-challenge with colonization level as high as 10^9^ CFU per gram of feces. In contrast, the isogenic *Cj0843c* mutant displayed significantly reduced colonization in the intestine (*P* < 0.05); the mutant was not detected in most of chickens. Complementation with the plasmid did not rescue the impaired colonization of the *Cj0843c* mutant, likely due to instability of the plasmid in the complemented strain. To test this, an *in vitro* plasmid stability assay was performed by subculturing complemented strain every 2–3 days in fresh antibiotic-free MH broth (1:400 dilution) for 10 days; by day 10, majority of the cells (85%) were observed to lose the complemented plasmid.

## Discussion

In this study, random transposon mutagenesis was used to identify novel genes contributing to beta-lactam resistance in *C. jejuni*, which led to the discovery of a putative LT Cj0843c that plays an important role in the intrinsic and acquired beta-lactam resistance in *C. jejuni*. Notably, the mutation of Cj0843c significantly reduced beta-lactamase activity and MIC for ampicillin (**Table 3** and **Figure 1**). The periplasmic Cj0843c was prevalent and highly conserved in *C. jejuni* and other 𝜀-proteobacteria, such as *C. coli* and *H. pylori* (**Supplementary Figure S1** and **Supplementary Table S1**), indicating the critically conserved role of Cj0843c in cell wall metabolism. Cj0843c is also required for *C. jejuni* colonization in the intestine (**Figure 2D**). Mutation of another cell wall metabolism-related gene pgp1 was also observed to impair *C. jejuni* colonization (Frirdich et al., 2012). However, at this stage, it is still premature to speculate how Cj0843c is involved in colonization. Mutation of Cj0843c may affect *C. jejuni* physiology and exert pleiotropic effects that are related to *C. jejuni in vivo* colonization.

The LT also have been observed to be involved in beta-lactam resistance in other bacteria. For instance, single (ΔMltB), double (ΔMltA/ΔMltB; Kraft et al., 1999) or sextuple (Slt70, MltA, MltB, MltC, MltD, and EmtA; Korsak et al., 2005) LT mutants in an *E. coli* MC1061 displayed significantly decreased beta-lactamase activity. Specific inhibition of the LT Slt70 by bulgecin A suppressed the activity of beta-lactamase AmpC in *E. coli* (Kraft et al., 1999). Notably, not all PG-degrading enzymes are involved in beta-lactam resistance. For instance, the deletion of two amidases (cleaving the amide bond between *N*-acetylmuramic acid and L-Ala) and three DD-endopeptidases (acting on the peptide cross-links) had little effect on beta-lactam resistance (Korsak et al., 2005). In *C. jejuni*, inactivation of Pgp1, which is involved in cleaving monomeric tripeptides to dipeptides (Frirdich et al., 2012), did not affect beta-lactamase activity and the susceptibility of *C. jejuni* to ampicillin as shown in this study.

The LT-mediated beta-lactam resistance identified in this study indicates LT is a promising target for combinational antimicrobial chemotherapy in *C. jejuni*, even in 𝜀-proteobacteria. Our genome survey suggested the three-gene operon containing Cj0843c is conserved across 𝜀-proteobacteria. The LT-mediated beta-lactam resistance mechanism might be also prevalent in 𝜀-proteobacteria. For example, the *H. pylori* homolog (Slt) shares high homology to the Cj0843c (31% aa identity and 51% aa similarity). Recently, Slt also has been found to contribute to amoxicillin resistance in *Helicobacter* (Bonis et al., 2012). Bulgecin A, the specific inhibitor of *H. pylori* Slt, could enhance the antimicrobial effect of amoxicillin (Bonis et al., 2012). Given amoxicillin is an important β-lactam component of the triple therapy to eradicate *H. pylori* infection in human (Tepes et al., 2012), LT inhibitor may serve as a good combination agent (e.g., together with beta-lactam/beta-lactamase inhibitors) to maximize the clinical efficacy of beta-lactam antibiotics.

The decreased β-lactamase activity in LT mutants might be correlated with altered metabolism of peptidoglycan (Kraft et al., 1999). In Gram-negative bacteria, the LT-degraded PG fragments, also called muropeptides, not only serve as cell wall recycling materials during cell growth, but also serve as a signal to turn on the production of β-lactamase through an intracellular transcriptional regulator (Jacobs et al., 1997; Boudreau et al., 2012; Zeng and Lin, 2013). Specifically, muropeptides was either transported into cytoplasm through inner membrane transport AmpG and bound by the regulator AmpR, or directly bound by response regulators of the BlrAB-like two-component regulatory system, to activate the expression of β-lactamase AmpC (Boudreau et al., 2012; Zeng and Lin, 2013). Mutation of the putative LT Cj0843c likely disrupt such intracellular signaling pathway, therefore interfering the induced expression of beta-lactamase and leading to significantly reduced beta-lactamase activity as well as ampicillin MIC observed in this study (**Figure 1**). This speculation needs to be examined in future studies.

## Author Contributions

JL is the project leader who oversights all the experiments described in this manuscript. XZ and JL contributed with the conception and design of the study. XZ and BG were involved in the collection of data. XZ and JL were involved in the analysis and interpretation of data; drafting and revision of the article; and final approval of the article.

## Conflict of Interest Statement

The authors declare that the research was conducted in the absence of any commercial or financial relationships that could be construed as a potential conflict of interest.

## Footnotes

1 https://img.jgi.doe.gov/cgi-bin/imgm_hmp/main.cgi

2 http://www.ebi.ac.uk/Tools/msa/clustalo/
