# Supplementary material for: Important Role of a Putative Lytic Transglycosylase Cj0843c in β-Lactam Resistance in Campylobacter jejuni
Source: Front Microbiol. 2015 Nov 17;6:1292. doi: 10.3389/fmicb.2015.01292 (PMC4647113; doi:10.3389/fmicb.2015.01292)
Supplement: Supplementary file 1 [file Presentation_1.PDF]

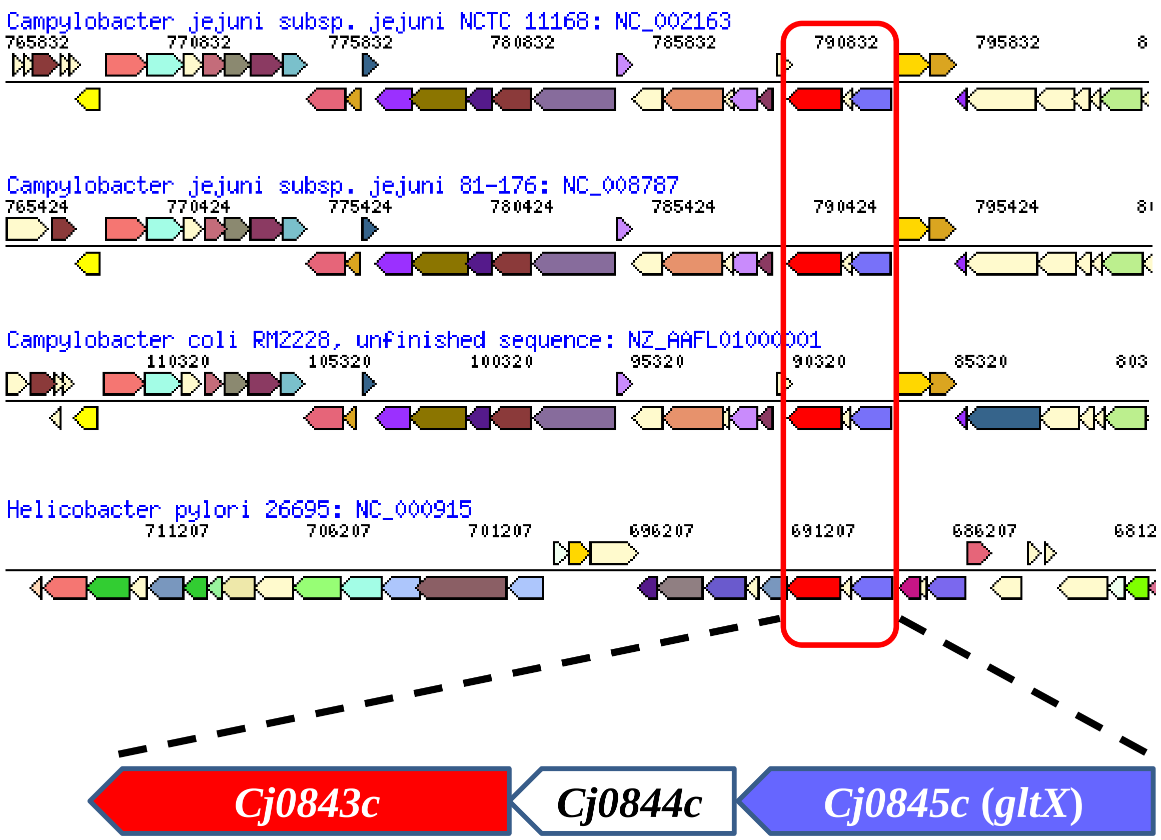

Figure S1. Cj0843c locus is conserved among *C. jejuni*, *C.coli* and *H. pylori*. *C. jejuni* NCTC 11168 and 81-176, *C. coli* RM2228 and *H. pylori* 26695 were chosen as representative strains. The 3-gene operon containing *Cj0843c* is circled in red rectangle.

Table S1. The presence of *Cj0843c* operon in available genomes of *C. jejuni*, *C. coli* and *H. pylori*.

C. jejuni		***C. coli***	***H. pylori***					
Cj1	S3	80352	8A3	BCS100H1	Hp A-4	wls-5-10	Rif2	GAM260BSi
1997-4	LMG 9879	RM1875	R037c	Hp H-9	GAM105Ai	HPAG1	R038b	Hp H-23
1577	2008-831	RM2228	HLJHP253	G27	FAM120Ai	v225d	Rif1	Hp H-34
1893	2008-872	H56	HLJ039	B8	GAM119Bi	83	Aklavik86	Hp H-24b
P110B	H22082	1948	B128	F32	Hp H-36	HUP-B14	26695	Hp H-24c
RB922	6399	1909	N6	CPY1662	Hp H-27	R055a	R018c	Hp M1
20176	04199	37/05	BM012A	BM012S	HP250ASi	NQ4110	GAM112Ai	A45
Cj2	LMG 23211	202/04	BM012B	UM023	HP250AFiV	NQ4053	CPY6311	GAM244Ai
g113	1928	2553	H13-1	J99	GAMCHJS117Ai	MALT	GAM263BFi	HP260Bi
2008-1025	LMG 23263	LMG 23341	Shi417	Shi169	HP250AFiii	Hp H-45	Hp P-1	NQ4161
110-21	00-2538	LMG 23342	XZ274	R046Wa	HP250AFii	HLJHP271	Hp P-3	HP260BFii
PT14	00-2426	86119	NQ228	NAB47	GAM252Bi	Oki154	Hp P-4	GAM80Ai
00-2544	10227	H6	UM018	SNT49	GAM250T	Oki102	P79	GAM96Ai
IA3902	NCTC 11168	RM5611	Lithuania75	Puno135	GAM252T	CG-IMSS-2012	Hp H-5b	Hp H-16
1798	LMG 23223	H9	Aklavik117	R036d	Hp P-74	UMO65	GAM239Bi	Hp P-25
255	30318	CVM N29716	NQ4044	Hp A-9	CPY1313	Shi470	GAM245Ai	Hp H-1
51494	1997-14	317/04	NQ4216	CPY6261	GAM250AFi	51	GAM42Ai	Hp H-18
1997-10	87459	2685	CPY1124	CPY6271	HP250ASii	52	GAM93Bi	Hp M5
60004	LMG 23210	CVM N29710	CPY3281	Hp A-11	HP250BFi	SJM180	Hp A-26	Hp M4
LMG 9081	2008-979	LMG 9860	NAD1	Hp P-30	HP250BFii	F57 DNA	Hp H-42	Hp M3
M1	NW	Z163	NAK7	UM066	HP250BFiii	R32b	Hp P-23	Hp M2
10186	30286	90-3	UM034	UM038	HP250BFiV	NQ4076	Hp P-16	Hp M9
RM1221	K5	H8	wls-5-3	UM066	HP250BSi	NQ4200	Hp H-10	Hp M6
CG8486	CG8421	1098	UMO84	wls-5-8	CCHI 33	CPY6081	NCTC 11638	GAM117Ai
1213	129-258	7-1	wls-5-2	wls-5-15	GAMchJs106B	GAM265BSii	Hp P-1b	Sahul64
BJ-CJD101	81-176 DRH212	1961	E48	wls-5-1	Hp A-8	CPY1962	Hp P-3b	UM077
HB93-13	81-176 UMCW9	Z156	Oki128	Oki898	Hp P-26	GAM246Ai	Hp P-4d	UMO85
81-176	LMG 23357	111-3	BMO13A	Oki422	Hp P-15	Hp A-27	Hp P-4c	PZ5086
2008-894	2008-988	K3	wls-5-9	BMO13B	UMO37	Hp P-13	R056a	Hp H-21
327	HPC5	2688	Hp H-19	wls-5-7	wls-5-12	Hp P-13b	UMO67	Hp P-41
K1	260.94	2680	wls-5-18	NQ352	UMO37	Hp A-14	PZ5056	Hp P-2
LMG 9872	ICDCCJ07001	84-2	NQ 4191	NQ367	wls-5-5	NQ1671	UM111	GAM231Ai
1854	ATCC 49349	23344	HLJHP193	NQ392	wls-5-14	NQ1707	26695	GAM249T
269.97	DFVF1099	LMG 23336	HLJHP256	NQ4060	wls-5-17	NQ1712	P12	Hp A-20
CF93-6	81-176 UMCW7	K7	UMO54	UM298	wls-5-6	NQ315	B38	Hp P-2b
84-25	ICDCCJ07004	LMG 9853	UMO45	UM032	wls-5-13	X47-2AL	SouthAfrica7	GAM103Bi
51037	55037	RM4661	UM299	Oki 673	wls-5-11	Oki828	Puno120	GAM201Ai
15742	81116	59-2	F30	wls-5-4	wls-5-16	PZ5026	Hp A-5	Hp A-17
86605	ICDCCJ07002	1891	35A	Shi112	Sat464	98-10	GAM115Ai	GAM270ASi
LMG 9217	HN-CJD07035	1091	ELS37	Hp P-15b	India7	2017	NQ4099	Hp H-3
LMG 23269	NCTC 11168-BN148	LMG 9854	J166	Hp A-16	Cuz20	2018		PeCan4
53161	XY259	2548	Hp H-11	Hp H-6	908	PeCan18	GAM254Ai	GAM101Biv
D2600	04197	1148	GAM121Aii	UM007	F16 DNA	Hp P-8	GAM260ASi	GAMchJs114i
Cj3	LMG 23218	15-537360	GAM100Ai	UM114	GAM264Ai	Hp P-11	Hp H-30	Hp H-4
Cj5	P854	JV20	HP260ASii	PZ5004	Hp A-6	PZ5080	GAM210Bi	NQ1701
1997-11	87330	2692	HP260AFi	J166	SouthAfrica20	Hp P-28b	Hp P-8b	Hp P-62
305	00-2425	1957	HP260AFii	GAM71Ai	Hp P-25c	Hp H-41	PZ5024	Hp H-28
414	23264	1417	GAM268Bii	Hp P-11b	GAM114Ai	GAMchJs1361	R030b	Hp H-29
1336	1997-7	151-9	GAM83T	Gambia94/24	HP116Bi		Hp H-24	SouthAfrica50
140-16	ATCC 33560		GAM83Bi	GAM118Bi			GAM260Bi	
1997-1			Hp H-44	GAMchJs124i				
